# Phylogenetic characterization of *East African cassava mosaic begomovirus* (*Geminiviridae*) isolated from *Manihot carthaginensis* subsp. *glaziovii* (Müll.Arg.) Allem., from a non-cassava growing region in Tanzania

**DOI:** 10.5897/AJB2017.16130

**Published:** 2017-09-06

**Authors:** F. Tairo, W. K. Mbewe, D. Mark, M. Lupembe, P. Sseruwagi, J. Ndunguru

**Affiliations:** 1 Mikocheni Agricultural Research Institute, P.O Box 6226, Dar es Salaam, Tanzania; 2 School of Agricultural Sciences, Makerere University, P. O. Box 7062, Kampala, Uganda; 3 Bvumbwe Agricultural Research Station, P. O. Box 5748, Limbe, Malawi

**Keywords:** Cassava mosaic disease, East African cassava mosaic virus, *Manihot carthaginensis* subsp. *glaziovii* (Müll.Arg.) Allem., genetic diversity

## Abstract

*Manihot carthaginensis* subsp. *glaziovii* (Müll.Arg.) Allem., a wild relative of cassava, native to Brazil, is one of the popular agroforestry trees used for hedges and/or boundary plants surrounding homesteads and farms and also harbours cassava mosaic begomoviruses (CMBs) and cassava brown streak ipomoviruses. Sequences of the DNA-A component of *East African cassava mosaic virus* (EACMV) isolates from *M. carthaginensis* subsp. *glaziovii* (Müll.Arg.) Allem., collected from non-cassava growing areas of Tanzania were characterized. Thirteen full length DNA-A sequences were analysed together with 15 already reported EACMV sequences and six CMB species reference genomes. The results show 96 to 100% nucleotide sequence identity with EACMV isolates from Kenya. Phylogenetic analysis revealed that EACMV isolates from *M. carthaginensis* subsp. *glaziovii (*Müll.Arg.) Allem, belong to a single cassava mosaic begomovirus species. The EACMV monophyletic clade is distinct from all other CMB species. The presence of Cassava infecting begomoviruses in wild cassava relative growing from traditionally non cassava growing region serve as inoculum sources for cassava-infecting begomoviruses and therefore their eradication is key in the sustainable management of CMBs, especially in the non-cassava growing areas.

## INTRODUCTION

*Manihot carthaginensis* subsp. *glaziovii* (Müll.Arg.) Allem., commonly called „tree cassava plays a crucial role in cassava improvement programs as the source of disease resistance, particularly for cassava mosaic disease (CMD) and cassava brown streak disease (CBSD) (Nichols, 1947). In traditional cassava growing areas, it provides cheap source vegetable while in non-cassava producing areas, it is one of the popular agroforestry trees used for hedge/or boundary plants surrounding homesteads and farms; it is also used in small quantities for animal fodder. However, despite its crucial roles, it is also responsible for the perpetuation of CMD and CBSD in traditional cassava growing areas and non-growing areas.

Several studies on the epidemiology of CMD have established a potential role of non-cassava plant species as alternate reservoir in perpetuation of CMBs (Alabi et al., 2008). However, these studies have revealed occurrence of at least three CMBs species in wild relative and weed plants, and each study focused primarily on traditional cassava growing areas. In Nigeria, both *African cassava mosaic virus* (ACMV) and *East African cassava mosaic virus* (EACMV) were reported in *M. carthaginensis* subsp. *glaziovii* (Müll.Arg.) Allem., and leguminous plants and in *Leucaena leucocepohala* (Alabi et al., 2008). But most of these studies have concentrated on traditional cassava growing areas where the interaction of cassava and its wild relatives and/or weeds is common. While some information is available on the natural occurrence of EACMV (Ogbe et al., 2006) in *Manihot* spp., little is known about the occurrence of cassava mosaic like symptoms in *M. carthaginensis* subsp. *glaziovii* (Müll.Arg.) Allem.*,* in the traditional non-cassava growing areas.

Kilimanjaro region in northern Tanzania is a traditional non cassava growing region, where *M. carthaginensis* subsp. *glaziovii* (Müll.Arg.) Allem., is a popular agroforestry tree used as a hedge/or border plant-surrounding banana and coffee fields. Significant part of the region is in lower land with favourable climate for commercial cassava production. Thus, understanding the status of CMD and the diversity of associated viruses is worth studying in order to devise a sustainable measure to eradicate the inoculum and a measure for the CMD sources. In this study, a total of 13 CMB DNA-A sequences from *M. carthaginensis* subsp. *glaziovii* (Müll.Arg.) Allem., sampled from non-cassava growing farmer fields in Kilimanjaro, Tanzania were characterized to investigate their identity and diversity in relation to corresponding DNA-A sequences.

## MATERIALS AND METHODS

**Figure 1:**
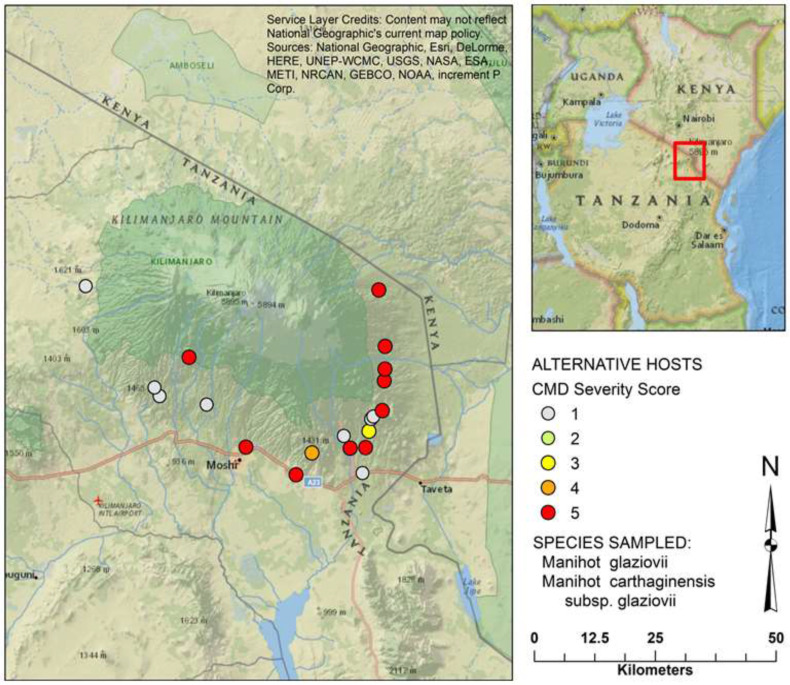
Map of Tanzania showing sampling areas.

**Figure 2:**
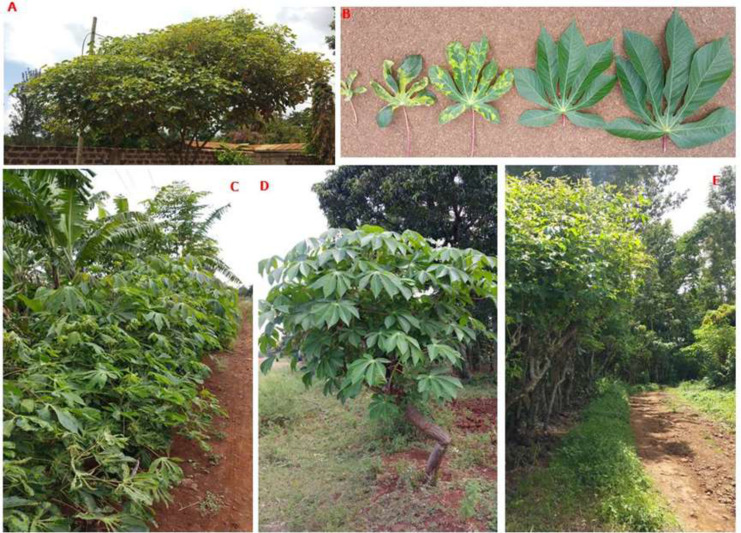
**A,** *M. carthaginensis* subsp. *glaziovii* (Müll.Arg.) Allem., growing in homesteads as shade trees. **B**, leaves with CMD-like symptoms. **C** and **E**, border surrounding a banana field. **D,** healthy plant along the road.

*M. carthaginensis* subsp. *glaziovii* (Müll.Arg.) Allem., leaf samples displaying cassava mosaic like symptoms ranging from mild chlorotic mosaic to severely distorted leaf and filform (Figure 2) were collected in 3 districts: Moshi rural, Rombo and Siha (Figure 1). Total DNA was extracted from leaves stored in a silica gel as described (Alabi et al., 2008) and used as a template for rolling-circle amplification (RCA) of complete begomovirus genomes as per Illustra TempliPhi amplification kit (GE Healthcare Life Sciences, UK). The RCA products were first PCR-amplified using begomovirus universal primer pair EBB555F /R1 (Fondong et al., 2000) to see if they contain any begomovirus infection, and subsequently used to construct Illumina libraries and sequenced at North Carolina State Genomic Sciences Laboratory (Raleigh, NC, USA) by next generation sequencing.

**Figure 3:**
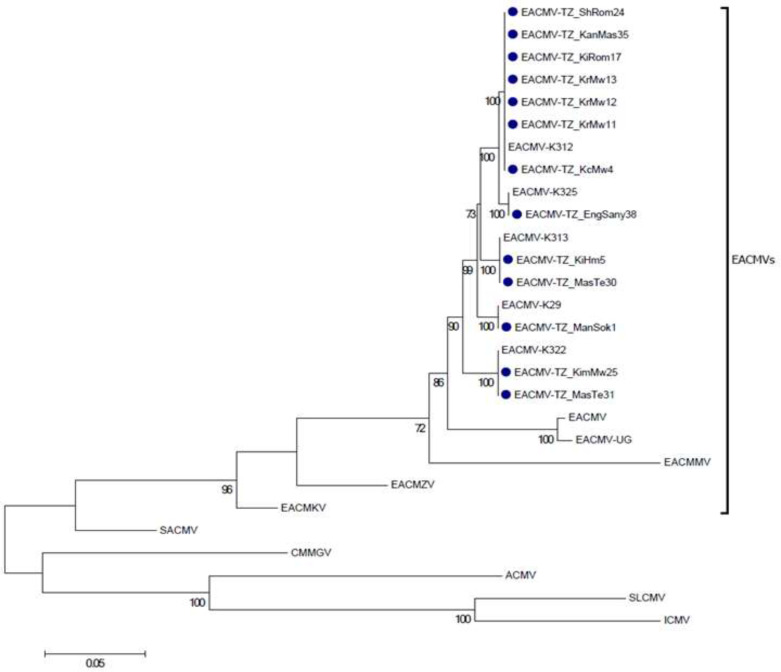
A ML phylogenetic tree of the complete sequences of EACMV-DNA-A isolated from *M. carthaginensis* subsp. *glaziovii* (Müll.Arg.) Allem., in Tanzania. Numbers in branches represent bootstrap values of 1000 replicates (shown only when >70%) and scale bar indicates nucleotide substitutions per site. The EACMV virus sequenced sequenced from *M. carthaginensis* subsp.*glaziovii* (Müll.Arg.) Allem., host in this study are shown in blue and abbreviated as Mg; EACMV[TZ:Kch:Mg4:17] *East African cassava mosaic virus* [Tanzania:Kichuimwika:2017], EACMV[TZ:Kih:Mg5:17] *East African cassava mosaic virus* [Tanzania:KilachaHimo:2017], EACMV[TZ:Krm:Mg11:17] *East African cassava mosaic virus* [Tanzania:KirueniMwika:2017], EACMV[TZ:Krm:Mg12:17] *East African cassava mosaic virus [Tanzania:*Kiruenimwika:2017], EACMV[TZ:Krm:Mg13:17] *East African cassava mosaic virus* [Tanzania:Kiruenimwika:2017], EACMV[TZ:Kel:Mg17:17], *East African cassava mosaic virus* [Tanzania:Kelamfuarombo:2017], EACMV[TZ:Shi:Mg24:17], *East African cassava mosaic virus* [Tanzania:Shimbirombo:2017], EACMV[TZ:Kim:Mg25:17] *East African cassava mosaic virus* [Tanzania:Kimangaro:2017], EACMV[TZ:Mas:Mg30:17] *East African cassava mosaic virus [Tanzania:*Masama:2017], EACMV[TZ:Mas:Mg31:17] *East African cassava mosaic virus* [Tanzania:Masama:2017] EACMV[TZ:Kan:Mg35:17] *East African cassava mosaic virus* [Tanzania:KangeriMashati:2017], EACMV[TZ:Eng:Mg38:17] *East African cassava mosaic virus* [Tanzania:Engarenairobi:2017], EACMV[TZ:Mam:Mg1:17], *East African cassava mosaic virus* [Tanzania:Mamsera:2017], EACMV-[K29] *East African cassava mosaic virus* [Kenya:K29], EACMV[K312] *East African cassava mosaic virus* [Kenya:K312], EACMV[K322] *East African cassava mosaic virus* [Kenya:K322], *East* EACMV[K325] *African cassava mosaic virus* [Kenya:K325], EACMV[K313] *East African cassava mosaic virus* [Kenya[K313]. Naming of isolates is based on Brown et al. (2015).

Raw reads for each sample were assembled using *de novo* assembly tool on CLC Genomics Workbench, mapped and aligned using reference sequences obtained from GenBank (Table 1 and Figure 3) representing full length DNA-A component of cassava begomoviruses under the following conditions: minimum overlap (10%), minimum overlap identity (80%), allow gaps (10%) and fine tuning set to iterate up to 10 times.

**Table 1:** Cassava mosaic begomovirus sequences used in the analysis.

Isolate name	Component	Location name	Accession number
EACMV-TZ_Kch:Mg4:17	DNA-A	Kichui Mwika, Moshi rural	MF067253
EACMV-TZ_Kih:Mg5:17	DNA-A	Kilacha Himo, Moshi rural	MF067254
EACMV-TZ_Krm:Mg11:17	DNA-A	Kirueni Mwika, Moshi rural	MF067255
EACMV-TZ_Krm:Mg12:17	DNA-A	Kirueni Mwika, Moshi rural	MF067256
EACMV-TZ_Krm:Mg13:17	DNA-A	Kirueni Mwika, Moshi rural	MF067257
EACMV-TZ_Kel:Mg17:17	DNA-A	Kilamfua, Rombo	MF067258
EACMV-TZ_Sh:Mg24:17	DNA-A	Shimbi, Rombo	MF067259
EACMV-TZ_Kim:Mg25:17	DNA-A	Kimangaro Mwika, Moshi rural	MF067260
EACMV-TZ_Mas:Mg30:17	DNA-A	Masama Tema, Siha	MF067261
EACMV-TZ_Mas:Mg31:17	DNA-A	Masama Tema, Siha	MF067262
EACMV-TZ_Kan:Mg35:17	DNA-A	Kangeri Mashati, Rombo	MF067263
EACMV-TZ_Eng:Mg38:	DNA-A	Engarenairobi Sanyajuu, Siha	MF067264
EACMV-TZ_Man:Mg1:17	DNA-A	Mansera Sokoni, Rombo	MF067265
EACMV-K29	DNA-A	Kwale Kibaoni, Kenya	AJ717551
EACMV-K312	DNA-A	Machakos Migwani, Kenya	AJ717547
EACMV-K322	DNA-A	Kwale kibaoni, Kenya	AJ717556
EACMV-K325	DNA-A	Kitui Township, Kenya	AJ717548
EACMV-K313	DNA-A	Machakos Migwani, Kenya	AJ717549
EACMMV	DNA-A	Malawi	AJ006460
EACMKV	DNA-A	Machakos,Kenya	AJ717571
EACMZV	DNA-A	Kwale Msambweni, Kenya	AJ717568
EACMV	DNA-A	Uganda	AF126806
EACMV-UG	DNA-A	Uganda	FN668377
SACMV	DNA-A	South Africa	AF1558061
CMMGV	DNA-A	Toliary, Madagascar	HE617300
ACMV	DNA-A	Pwani,Tanzania	AY795982
SLCMV	DNA-A	Kerala, India	AJ890226
ICMV	DNA-A	Kerala, India	AJ575820

Nucleotide sequence identities were computed using sequence demarcation tool (SDT) version 1.2 (Muhire et al., 2014). The identity scores were calculated as 1-(M/N) where M is the number of mismatching nucleotides and N the total number of positions along the alignment at which neither sequence has a gap (Muhire et al., 2014). Multiple sequence alignments of the full length DNA-A component determined from *M. carthaginensis* subsp. *glaziovii* (Müll.Arg.) Allem., were generated using the Clustal W alignment function in Mega 7 (Kumar et al., 2016) and edited visually. Same MEGA 7 was used to construct maximum-likelihood (ML) phylogenetic trees. Initial trees for the heuristic search were obtained automatically by applying Neighbor-Joining algorithm (Tamura et al., 2004) to a matrix of pairwise distances estimated using the maximum composite likelihood approach, and then selecting the topology with superior log likelihood value (Kumar et al., 2016). All positions containing gaps and missing data were eliminated. Evolutionary analyses were conducted in MEGA7 (Kumar et al., 2016). The General Time Reversible (GTR) nucleotide substitution model was used (selected as the most appropriate by ML). The stability of the inferred branches was estimated by bootstrapping with 1000 replicates.

## RESULTS AND DISCUSSION

PCR screening using begomovirus universal primer pair (Fondong et al., 2000) amplified expected fragments of 552 bp both in symptomatic and non-symptomatic samples indicating they were singly infected with begomovirus species. The next generation sequencing reads of 38 samples produced a pired sequences data of 7,247,392.00 million reads. After trimming for non-viral sequences 7,151,881 million reads remained and were assembled *de novo* to a total of 40 contigs. Subsequent Blast search of the assembled contigs identified 23 contigs ranging from 201 to 620 nt in length with average of 411 nt with respect to reference sequences in the GenBank from which 13 full length sequences (2,800 nts) corresponding to DNA A were obtained. The resulting nucleotide sequences were deposited in GenBank under accession numbers MF067253-MF067265 (Table 1).

Pairwise comparison of full-length sequences of DNA-A molecules with available sequences in GenBank suggested all the sequences are related to EACMV Kenyan isolates (Figure 3). The DNA-A sequences were the most similar (97 to 100% nt sequences identity) to EACMV-Kenyan isolates as compared to DNA-B with 92 to 100% nt sequence identity. Based on begomovirus thresholds for species demarcation (Brown et al., 2015), a phylogenetic tree based on the DNA-A component sequences demonstrated a close genetic relationship among EACMV isolated from *M. carthaginensis* subsp. *glaziovii* (Müll.Arg.) Allem., in this study with EACMV Kenyan isolates (Figure 3).

A phylogenetic tree constructed using all 13 DNA-A full length sequences and those available in the GenBank revealed at least two major clusters, with the second cluster having at least four monophyletic clades (Figure 3). This result suggests that although all the EACMV isolates from *M. carthaginensis* subsp. *glaziovii* (Müll.Arg.) Allem. in this study clustered in at least four different clades, there is a still high level of similarity with nucleotide sequence identity between clades, with high similarity in the nucleotide sequence identity of 98 and 99%.. Search for any evidence of recombination among the sequences of full length DNA-A components isolated in this study using RDP4 analysis revealed no evidence of any recombination event.

CMD is a serious problem in SSA, especially in a traditional cassava growing regions, where CMD inoculums and whitefly (*Bemisia tabaci*) vector populations appear to be high throughout the year. Identification of EACMV in *M. carthaginensis* subsp. *glaziovii* (Müll.Arg.) Allem. in three districts in the slope of Mt. Kilimanjaro around 1,573 m above sea level in Tanzania shows CMB inoculum is already present in the region ahead of cassava cultivation. Thus, earlier reports on the factors influencing perpetuation of CMD were confirmed (De Bruyn et al., 2016). Analysis of nucleotide sequences of full length DNA-A and absence of recombination among the determined sequences showed that only one species of CMB is restricted within the surveyed districts with possible introduction from the nearby country (Figures 1 and 3), and continues to spread through use of infected cuttings. Since *M. carthaginensis* subsp. *glaziovii* (Müll.Arg.) Allem. in Kilimanjaro region is an important agroforestry tree, there is a need to creat awareness on its role in introducing and spreading cassava mosaic begomoviruses and cassava brown streak viruses. There is no doubt that continuous use of virus-infected *M. carthaginensis* subsp. *glaziovii* (Müll.Arg.) Allem., as hedge/or border plants guarantees the most efficient virus inoculum reservoir for introduction of CMD into cassava once introduced in the region. The findings form the basis for strategic management and possible eradication of the CMD-affected plants of M. *carthaginensis* subsp. *glaziovii* (Müll.Arg.) Allem., as inoculum sources. This may be achieved through conducting aggressive awareness campaigns to educate farmers on CMD epidemiology coupled with eradication of all plants with CMD-like symptoms to limit further spread of CMD. It is therefore recommended that, given availability of resources, cassava viral disease surveillance should not be limited to traditionally cassava growing regions only.
